# Supplementary material for: In vivo assessment of mechanisms underlying the neurovascular basis of postictal amnesia
Source: Sci Rep. 2020 Sep 14;10:14992. doi: 10.1038/s41598-020-71935-6 (PMC7490395; doi:10.1038/s41598-020-71935-6)
Supplement: Supplementary file 1 — Supplementary Information. [file 41598_2020_71935_MOESM1_ESM.pdf]

Supplementary Material to:

In vivo assessment of mechanisms underlying the neurovascular basis of postictal amnesia

Jordan S. Farrell[1,2#*], Roberto Colangeli[2#], Barna Dudok[1], Marshal D. Wolff[2], Sarah L. Nguyen[3], Jesse Jackson[4,5], Clayton T. Dickson[3-5], Ivan Soltesz[1], G. Campbell Teskey[2]

1. Department of Neurosurgery, Stanford University, Stanford, CA, USA
2. Hotchkiss Brain Institute, University of Calgary, Calgary, AB, Canada
3. Department of Psychology, University of Alberta, Edmonton, AB, Canada
4. Department of Physiology, University of Alberta, Edmonton, AB, Canada
5. Neuroscience and Mental Health Institute, University of Alberta, Edmonton, AB, Canada

*Corresponding author and lead contact: J.S.F. jsfarrel@stanford.edu
[#]Authors contributed equally to this work

Supplementary Figures

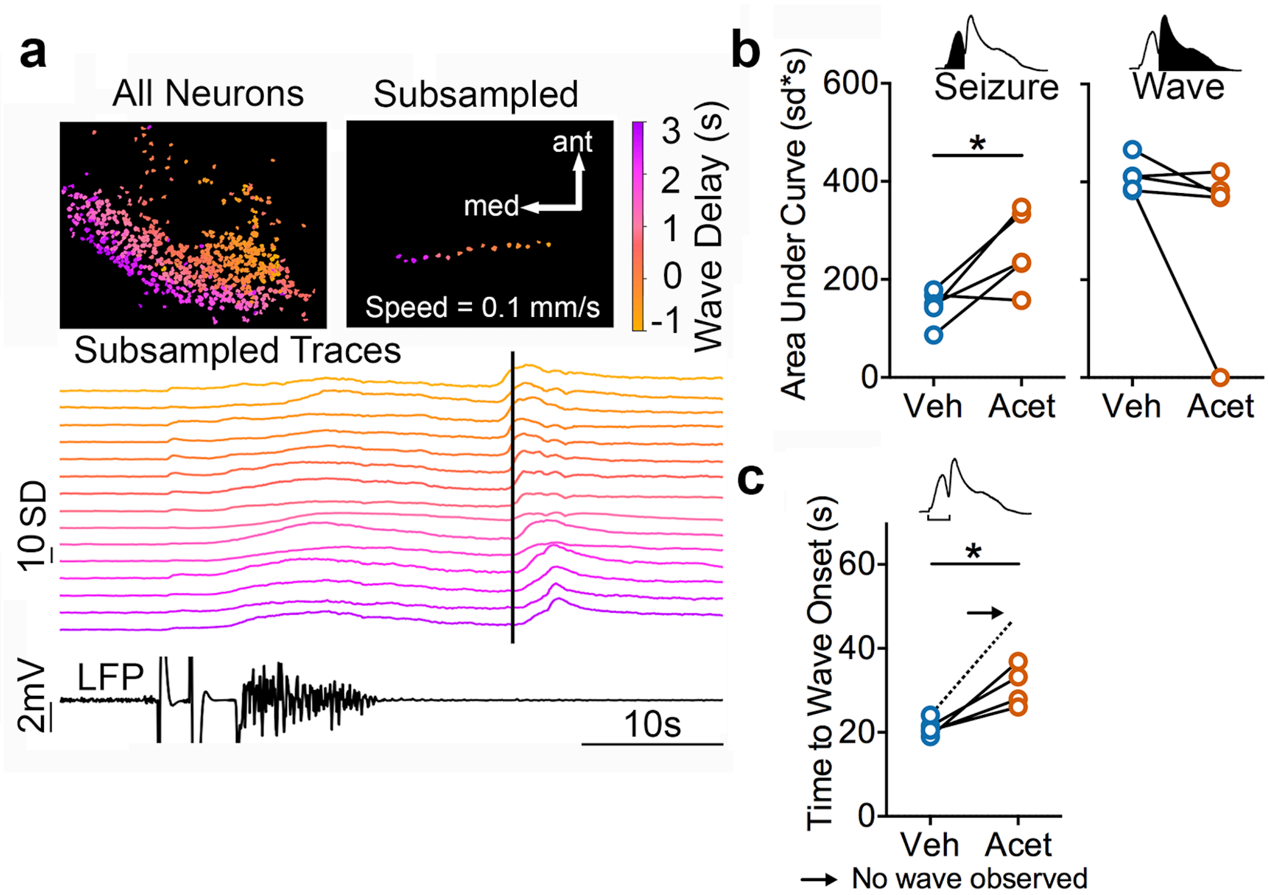

Supplementary Figure 1: Seizure vs. wave dynamic alterations by acetaminophen

(**a**) Data from Figure 1c plotted to demonstrate spreading wave propagation direction and speed. For each recorded neuron, the color displayed corresponds to the delay of wave onset relative to the mean wave onset. Individual calcium traces from an unbiased subsampled group of 15 neurons show the direction of wave propagation travels towards the medial and posterior edges of the field of view. Arrows indicate medial and anterior directions. Vertical black bar indicates mean wave onset. (**b**) Quantification of calcium dynamics during seizures and calcium waves. Acetaminophen increased the area under the curve during the seizure (left; $t(4)=3.14$, $p=0.03$, paired t-test), but not the secondary wave (middle; $t(4)=1.41$, $p=0.23$). Y-axis applies to both graphs. (**c**) Acetaminophen significantly delayed the onset of the calcium wave appearance from seizure onset (paired t-test, $t(3)=3.93$, $p=0.03$. In one case (arrow, dotted line), no calcium wave appeared (data not included in analysis), which is also evident in the top and middle plots.

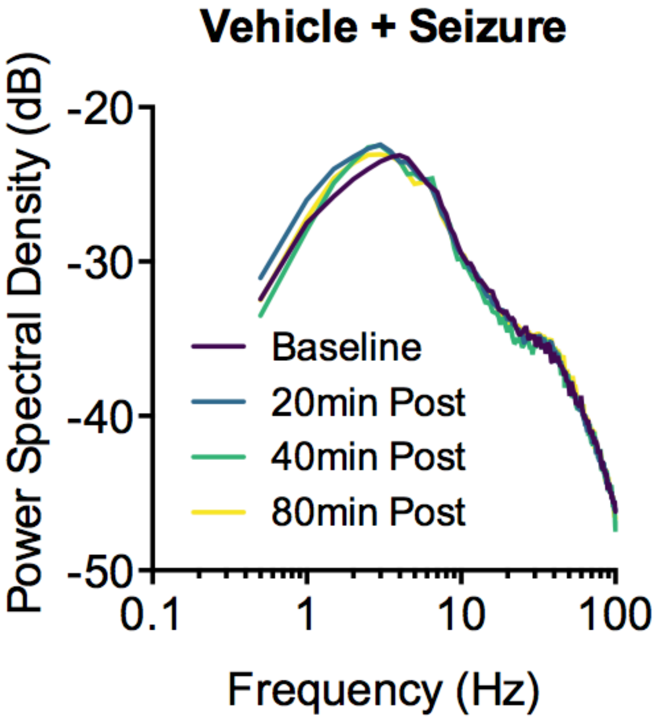

Supplementary Figure 2: Postictal vasoconstriction does not alter the local field potentials in awake mice
Mean power spectral density plots are displayed before and 20, 40, or 80 minutes after a seizure. LFP was recorded as a differential recording between the two poles (tips 0.5 mm apart) of the simulating/recording electrode. Repeated measures ANOVA did not find an effect of time at the 0-4, 4-12, 25-55, and 55-100 Hz frequency bands.

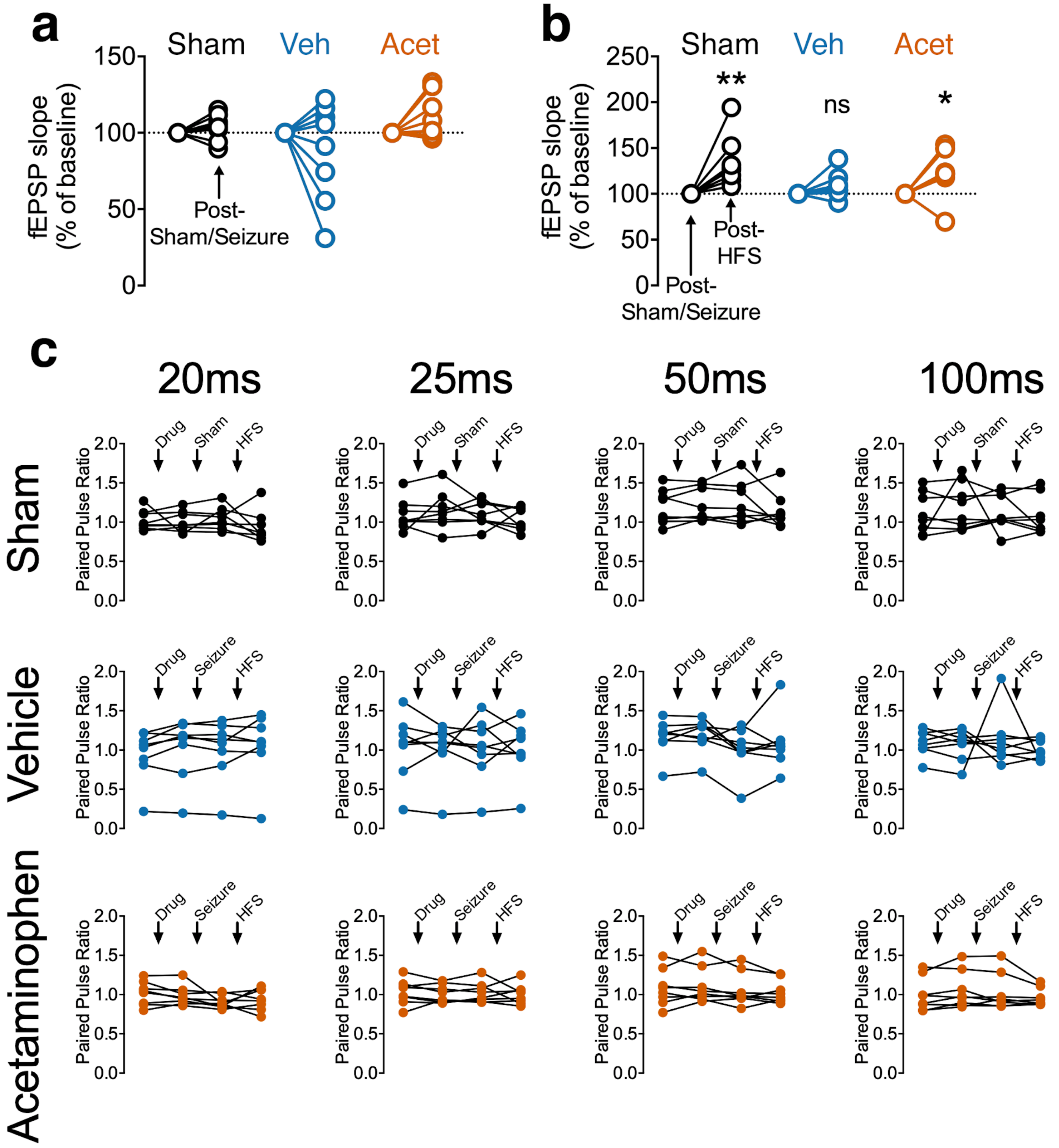

Supplementary Figure 3: Seizures do not disrupt evoked responses or paired-pulse ratios

(**a**) No significant changes in fEPSP slope were observed following kindling stimulation (or sham) (sham in black - t(7)=1.21, p=0.27; vehicle in blue - t(7)=1.03, p=0.27; acetaminophen in orange - t(7)=1.96, p=0.09). (**b**) Data from Fig. 3c normalized to a baseline beginning after the seizure or sham. Significant potentiation was observed in sham controls (black - t(7)=3.60, p=0.0092) and rats who received acetaminophen prior to seizure induction (orange - t(7)=2.49, p=0.042), whereas no significant potentiation was observed in rats that received vehicle prior to seizure (blue - t(7)=1.81, p=0.11). (**c**) The paired pulse ratio from individual rats are plotted in a matrix according to the interval between the first and second pulse (20 -100ms) and treatment (Sham, Vehicle, or Acetaminophen). Paired one-way ANOVAs did not find a significant difference for any of the intervals or treatments.

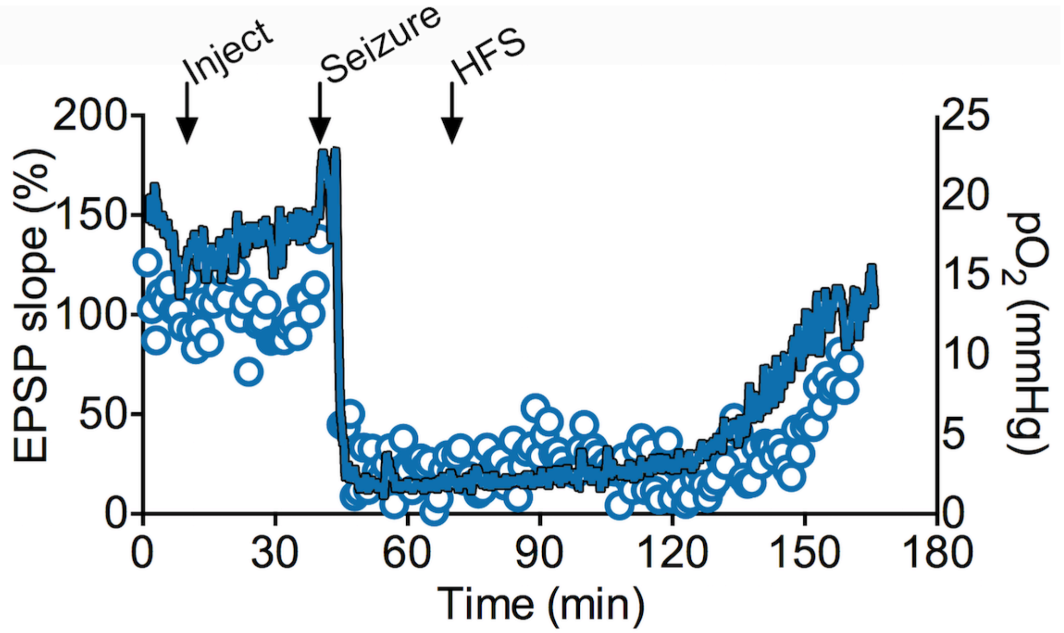

Supplementary Figure 4: One exceptional example of suppression of evoked potentials following a seizure An uncharacteristic example of a rat that experienced the most severe hypoxia in the experiment. The minimum pO_2 reached 1.2mmHg and oxygen levels remained severely hypoxic (<10mmHg) for 102.8 minutes. Though LTP could not be induced, like other rats, a major reduction in evoked responses was uniquely noted during the entire postictal period. Note that the oxygen profile recovered before the evoked response recovered.
